# SNPs in microRNA target sites and their potential role in human disease

**DOI:** 10.1098/rsob.170019

**Published:** 2017-04-05

**Authors:** Adrianna Moszyńska, Magdalena Gebert, James F. Collawn, Rafał Bartoszewski

**Affiliations:** 1 Department of Biology and Pharmaceutical Botany, Medical University of Gdansk, Gdansk, Poland; 2 Department of Cell, Developmental and Integrative Biology, University of Alabama at Birmingham, Birmingham, AL, USA

**Keywords:** poly-miRTS, microRNA, single nucleotide polymorphisms

## Abstract

In the post-genomic era, the goal of personalized medicine is to determine the correlation between genotype and phenotype. Developing high-throughput genotyping technologies such as genome-wide association studies (GWAS) and the 1000 Genomes Project (http://www.internationalgenome.org/about/#1000G_PROJECT) has dramatically enhanced our ability to map where changes in the genome occur on a population level by identifying millions of single nucleotide polymorphisms (SNPs). Polymorphisms, particularly those within the coding regions of proteins and at splice junctions, have received the most attention, but it is also now clear that polymorphisms in the non-coding regions are important. In these non-coding regions, the enhancer and promoter regions have received the most attention, whereas the 3′-UTR regions have until recently been overlooked. In this review, we examine how SNPs affect microRNA-binding sites in these regions, and how mRNA stability changes can lead to disease pathogenesis.

## 1. Introduction

Single nucleotide polymorphisms (SNPs) occur in 1% or more within the population [1]. Although these populations are identical in 99.5% at the DNA level [2], there are approximately 10 million SNPs in the human genome, indicating that they occur once in every 300 bp in both coding and non-coding regions of genes [3]. SNPs in the coding region can result in synonymous and non-synonymous changes, with the latter resulting in an amino acid change or the introduction of a stop codon [4]. These changes can lead to human diseases [5], and in fact at least 25% of the reported non-synonymous SNPs are predicted to negatively affect protein function [6,7].

Synonymous SNPs have been referred to as silent mutations because they do not change the amino acid [8]. However, there is a growing body of evidence indicating that synonymous SNPs do affect proper protein function [9]. For example, two synonymous SNPs in the sequence encoding the multidrug resistance protein 1 (MDR1) affect protein folding and function [10]. Moreover, the most common disease-causing mutation in the cystic fibrosis transmembrane conductance regulator (CFTR) gene is an out-of-frame deletion of phenylalanine-508 (ΔF508) that introduces a SNP at isoleucine-507 (I507) and this SNP contributes to the severity of the ΔF508 CFTR channel dysfunction [11,12].

Recently, more attention has been paid to the SNPs identified in non-coding regions. Interestingly, about 93% of functional SNPs in the GWAS catalogue are in non-coding regions [13]. They have been called regulatory SNPs (rSNPs) because they affect transcriptional regulation or post-transcriptional gene expression [14]. rSNPs can cause changes in cell function at different levels of gene regulation. For example, they can affect gene splicing [15] and transcription factor binding [16]. These rSNPs reside in the sequence of non-coding RNA in the promoter and enhancer regions [16]. They can also affect the half-life of messenger RNA (mRNA) and result in decreased protein levels through mRNA–microRNA (miRNA) interactions. SNPs in miRNA target sites in the 3′-UTR of mRNAs are referred to as poly-miRTSs [17]. The SNP dataset from the UCSC Genome browser (NCBI dbSNP, Build 130 [18]) consists of 18 833 531 human SNPs, while the genomic coordinates for a subset of 175 351 (approx. 11%) locates them in the 3'-UTRs of 16 810 genes [19]. Given that there are an estimated 19 000–20 000 genes in the human genome, this suggests that the majority of the genes could be regulated by miRNAs [20], indicating that the potential biological consequence of these mutations should be carefully considered. Furthermore, a substantial number of SNPs and rare mutations within pri-, pre- and mature miRNA sequences have been reported [21,22]. Although some of these miRNA SNPs are related to human diseases [23–27] (reviewed in [17]), their biological role is difficult to elucidate given that changes in any miRNA can have profound genome-wide effects since miRNAs can bind to hundreds of different mRNAs. Since 2008, when Sethupathy & Collins [17] critically reviewed reports of miRNA SNPs involved in human diseases and provided clear criteria for validation of such associations, a large number of novel human disease-related poly-miRTSs have been proposed. Furthermore, recently developed approaches dedicated to miRNA function, targeted genome editing with *in silico* methods provide novel tools for complex verification of miRNA SNP consequences. In this review, we focus on poly-miRTSs and their potential impact in human diseases.

## 2. SNPs in miRNA target sites

### 2.1. mRNA : miRNA association

miRNAs are short (approx. 22 nt) endogenous non-coding single-stranded RNAs which act as post-transcriptional regulators of gene expression [28]. In the cytosol, mature miRNAs that are a part of the Argonaute-containing silencing complexes called miRNA ribonucleoprotein complexes (miRNP) can downregulate a specific target mRNA by Argonaute-catalysed degradation of the mRNA target strand in P bodies or by translational repression [29,30]. Hence, the major consequence of miRNA : mRNA pairing is loss of protein expression, resulting from either decreased transcript levels or by translational repression [29].

Although the mechanism underlying the recognition of mRNA targets by miRNAs has been extensively studied, the minimal requirements for a functional mRNA : miRNA association are not fully understood. Furthermore, despite the fact that many mRNAs have conserved miRNA target sites, a variety of interactions through non-conserved sites has been reported [31]. Finally, the average size of the human 3′-UTR is about 950 nt (for highly expressed neuronal genes it is 1300 nt, whereas for genes specific to non-neuronal tissue it is only 700 nt) [32], while the efficient miRNA-binding site consists of 6–8 nt. Hence, the 3′-UTR of a specific mRNA can include tandem target sequences for a specific miRNA as well as target sequences for many other miRNAs. The composition of specific miRNAs associated with the 3′-UTR of a mRNA along with the efficiency of miRNA pairing to their target sequences impacts the mRNA's half-life and influences protein levels [33,34]. Hence, determining the consequences of SNPs in miRNA target sites is not a trivial endeavour.

**Figure 1.**
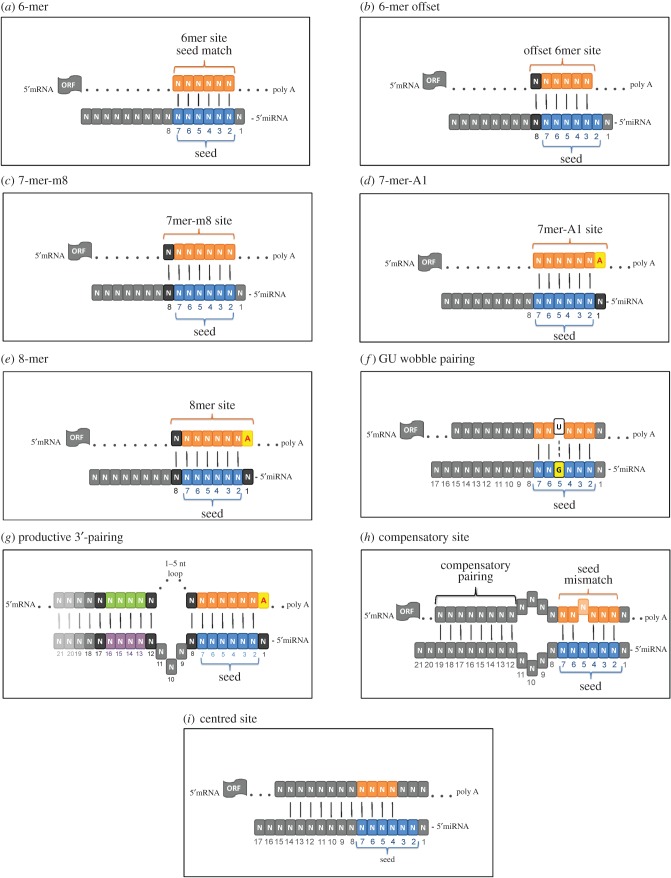
Types of mRNA : miRNA interactions. (*a*) 6-mer, (*b*) 6-mer offset, (*c*) 7-mer-m8, (*d*) 7-mer-A1, (*e*) 8-mer, (*f*) GU wobble pairing, (*g*) productive 3'-pairing, (*h*) compensatory site and (*i*) centred site.

That being said, it is well established that the complementary pairing of a 3′-UTR of a mRNA to a conserved heptametrical seed sequence is usually found at positions 2–7 from the miRNA 5′-end and is critical for mRNA target selection [35]. Initially, it was thought that perfect complementarity of the 3′-UTR of a mRNA to the miRNA seed sequence led to transcript degradation, and a partial match caused translational inhibition [35]. However, recent studies have shown that non-canonical sites also exist and can regulate mRNA degradation [36]. Furthermore, base pairing between mRNA and miRNA seed sequences do not always lead to decreased expression of target transcript [37]. The above findings suggest that additional features of mRNA target sequences play a crucial role in effective miRNA binding. The detailed analysis of seed sequences established 8-nt pairing (8-mer) with mRNA as the most effective, whereas 7- and 6-nt binding sites (7-mer and 6-mer) were less effective (figure 1). Although 6-mers often provide efficient pairing, even in an offset position (figure 1*a*,*b*), a 4-mer is a non-functional site *in vivo* [38]. Interestingly, 7-mer pairing efficiency relies strictly on sequence complementarity. Consequently, although the 7-mer-m8 site (an exact match to positions 2–8 of the mature miRNA—the seed and position 8 (figure 1*c*)) has increased seed pairing compared with the 6-mer, the 7-mer-A1 (an exact match to positions 2–7 of the mature miRNA—the seed followed by an ‘A1’) has similar seed pairing to 6-mer (figure 1*d*). The seed pairing including both the match at position m8 and the A1 is characteristic for a 8-mer site [37] (figure 1*e*). The effect of G : U base pairs and bulges in the seed were also considered showing that a single G : U wobble or target sites with a 1 nt bulge can still be functional [38] (figure 1*f*). However, the Watson–Crick base pairing is absolutely critical between nucleotides at positions 9–12 in the target site, since the hydrolysis of the phosphodiester backbone in mRNA cleaved by miRNA occurs only when the 10th and 11th nucleotides of mRNA are complementary to nucleotides at positions 2–15 in miRNA [39].

Furthermore, additional mRNA pairing to the 3′ region of miRNA, termed as productive seed pairing, can increase the target recognition or it can compensate for the mismatch to the seed (3′ supplementary sites and 3′ compensatory sites, respectively) [36]. The substantial pairing of 3′ compensatory sites to mRNA increases the weak 5′ pairing, resulting in functional miRNA binding (figure 1*g*,*h*).

Interestingly, Shin *et al*. [30] indicated that centred mRNA sequences consisting of 11 nt create Watson–Crick pairs with miRNA nucleotides at positions 4–14 or 5–15 and serve as a type of miRNA target site. This unique class of miRNA target sites is devoid of both perfect seed pairing and 3′ compensatory pairing but can be supplemented by pairing to the other miRNA areas (figure 1*i*).

Based on the studies discussed above, mRNA target sites can be divided into two major groups. The first group consists of canonical sites with (i) strong seed pairing to the 5′ end of miRNA (low pairing energy) that is amplified through either strong base pairing to the 3′ end of the miRNA (an extension of the seed type) or (ii) strong seed pairing to the 5′ end of miRNA seed sites requiring little or no 3′-UTR pairing support. These canonical sites have pairing energy and are often functional in one copy. In contrast with these sites, the second groups are non-canonical seed sites with higher pairing energy that should exist in the 3′-UTR in more than one copy to be effective [38]. It has to be stressed that the seed region contributes the majority of binding energy and strong binding relies mainly on base pairing within this region, whereas an additional 3′ pairing only slightly reduces binding energy [40]. Interestingly, pairing beyond position 16 and at positions 10–11 increases binding energy that results in weakened binding [40].

Another factor to consider in miRNA : mRNA interactions is the location of the target mRNA sites. In general, the 3′-UTR mRNA sites are most efficient [37,41]. Furthermore, target mRNA sites positioned within at least 15 nt from the stop codon, sites located away from the centres of long 3′-UTR, as well as those miRNA target sites located in AU-rich regions are the most effective [37,41]. Additionally, a location of target mRNA sites in close proximity to protein-binding sites and to other miRNA-binding sites may also affect miRNA : mRNA associations [33,37]. The 3′-UTR mRNA quartiles bordering the mRNA poly(A) tail and the ORF exhibit more effective targeting than remaining two centred quartiles. However, this effect was apparent only for longer 3′-UTRs (more than 1300 nt) [37].

Taking into account the complexity of miRNA : mRNA pairing, the introduction of a SNP into a 3′-UTR can have numerous functional consequences by either introducing or removing miRNA target sequences or changing the binding efficiency. The poly-miRTSs within the canonical seed sequence can either create a novel mRNA target site from a preexisting 5-mer sequence (into 6-mer offset or 6-mer) or impair the existing target site 6-mer or 6-mer offset sequence (into 5-mer). Furthermore, since the introduction of poly-miRTSs into seed regions can also affect miRNA : mRNA binding efficiency, it can lead to either increased or decreased post-transcriptional mRNA regulation. Finally, poly-miRTSs may also affect miRNA-binding efficiency by changing supplemental seed pairing that applies to both canonical and non-canonical binding sites. Additionally, in the case of non-canonical binding sites, poly-miRTSs may introduce or remove tandem target sites, and thus change the miRNA effects. Finally, the introduction or removal of miRNA target sites may affect binding to other miRNA target sequences in the SNP's close proximity, which could have unforeseen effects on the mRNA half-life. Given the number of SNPs in the human population, it is not surprising that poly-miRTSs have been shown to affect the levels of numerous proteins that have been associated with various disorders (table 1) [39]. Below, we discuss examples of several studies identifying poly-miRTSs and their potential association with human disorders.

**Table 1.** Reports of poly-miRTS associations with human disease. Bold indicates the studies fulfilled the criteria for assigning SNPs as poly-miRTSs involved in human diseases and included: (i) functional experimental validation of SNPs related to differential mRNA targeting; (ii) genetic testing of the association with the disease that takes into account the effects of population stratification; and (iii) mechanistic testing underlying the mechanism by which poly-miRTSs contribute to the disease [17].

associated disease or trait	miRNA	target gene	putative risk allele	functional association test for allele-specific effects on miRNA targeting	association test	population	refs
small cell lung cancer SCLC	miR-191, miR-887-3p	*MDM4*	rs4245739 A>C (C creates a new binding site)	*in vitro*: reporter gene assay in SCLCH446 cells (with miR mimics, or negative controls).	yes	Han Chinese	[42]
prostate cancer	miR-191, miR-887-3p	*MDM4*	rs4245739 A>C (C creates a new binding site)	*in vitro*: reporter gene assay in PC3 cells (with miR mimics, or negative controls).	none	—	[43]
**ovarian cancer**	**miR-191**	***MDM4***	**rs4245739 A>C (C creates a new binding site)**	***in vitro*: reporter gene assay in A2780 cells (with miR mimics). Target site and mismatch control blocker were used.**	**yes**	**Caucasian women**	[44]
non-Hodgkin lymphoma NHL	miR-191	*MDM4*	rs4245739 A>C (C creates a new binding site)	none	yes	Han Chinese	[45]
oesophageal squamous cell carcinoma ESCC	miR-191	*MDM4*	rs4245739 A>C (C creates a new binding site)	none	yes	Han Chinese	[46]
non-small cell lung cancer NSCLC	miR-887-3p	*MDM4*	rs4245739 A>C (C creates a new binding site)	*in vitro*: reporter gene assay in A549 cells (with miR mimics, or negative controls).	yes	Chinese	[47]
**bladder cancer**	**miR-140-5p**	***TP63***	**rs35592567 C>T (T creates a new binding site)**	***in vitro*: reporter gene assay in T24, EJ, 5637, J82 and 293A cells (with miR mimic or control).**	**yes**	**Han Chinese**	[48]
type 2 diabetes mellitus T2DM	miR-214-5p, miR-550a-5p	*HNF1B*	rs2229295 C>A (A creates a new binding site)	*in vitro*: reporter gene assay in HEK293 cells (with miR mimics).	yes	Japanese	[49]
**coronary heart disease**	**miR-4271**	***APOC3***	**rs4225 G>T (T creates a new binding site)**	***in vitro*: reporter gene assay in 293T and HepG2 cells (with miR mimic, inhibitor or control).**	**yes**	**Han Chinese**	[50]
hypertriglyceridaemia	miR-485-5p	*APOA5*	c.^a^*158C>T rs2266788 (rare c.^a^*158C allele creates a new binding site)	*in vitro*: luciferase expression vectors in 293T cells (with miR mimic, inhibitor or control); luciferase expression vectors in HuH-7 cells–investigating endogenous miR functionality (with miR inhibitor or control).	none	—	[51]
antropometrics (obesity related phenotype)	miR-522	*PLIN4*	rs8887 G>A (A creates a new binding site)	*in vitro*: luciferase expression vectors in COS7 cells (with miR mimic or control).	yes	Genetics of Lipid Lowering Drugs and Diet Network (GOLDN) and the Framingham Offspring Study (FOS).	[52]
Friedreich's ataxia FRDA	miR-124-3p	*FXN*	rs11145043 G>T (T creates a new binding site)	*in vitro*: reporter gene assay in HEK-293 cells (with miR mimic, or mimic negative control). Plasmid constructs differed in more than one SNP.	yes	paediatric cases (the Necker Children's Hospital), adult cases (the CHR Félix Guyon, Saint-Denis, La Réunion, France), controls (patients genetically tested at the Necker Children's Hospital for diseases non-related to FRDA).	[53]
Parkinson's disease	miR-34b	*SNCA*	rs10024743 T>G (unspecified)	*in vitro*: reporter gene assay in SH-SY5Y human neuroblastoma cells (with pre-miR or miR inhibitor; internal control). Immunocytochemistry using an anti-α-syn antibody.	none	—	[54]
**breast cancer**	**miR-96, miR-182**	***PALLD***	**rs1071738 C>G (G impairs binding site)**	***in vitro*: reporter gene assay in HeLa and HEK-293T cells (with miR mimic, miR inhibitor or control).**	**yes**	**460 homogeneous samples (Caucasians); study-sample: 68% Caucasians, 16% African-American, 6% Asian and 10% others**	[55]
schizophrenia	miR-137	*EFNB2*	rs550067317 A>C (C impairs binding site)	*in vitro*: reporter gene assay in HEK293T and SH-SY5Y cells (with miR mimic or negative control).	none	Han Chinese	[56]
**pancreatic ductal adenocarcinoma**	**miR-199a**	***HIF1A***	**rs2057482 T>C (C impairs binding site)**	***in vitro*: reporter gene assay in HEK293T and Panc-1 cells (with miR mimic, miR inhibitor or inhibitor control) *In vivo*: *HIF-1* expression in PDAC tissues (different genotypes).**	**yes**	**Han Chinese**	[57]
bladder cancer	miR-27b	*DROSHA*	rs10719 T>C (C impairs binding site)	*in vitro*: reporter gene assay in T24 and J82 cells (with miR mimic, stable negative control, miR inhibitor or inhibitor negative control), *In vivo*: analysis of total RNA in 61 bladder tumour tissues with different genotypes (32 for TT, 24 for TC, and 5 for CC).	yes	Han Chinese in Beijing (CHB)	[58]
**Parkinson's disease**	**miR-433**	***FGF20***	**rs12720208 C/T (T impairs binding efficiency)**	***in vitro*: reporter gene assay in Neuro2A cells (with miRNA mimic) *In vivo*: immunoblot analysis in three human brains with different genotypes.**	**yes**	**white Americans**	[59]
Tourette's syndrome	miR-189	*SLITRK1*	var321-SLITRK1 G>A (A creates Watson–Crick pairing instead of G:U wobble base pairing)	*in vitro*: reporter gene assay in Neuro2A mouse neuroblastoma cells (with miR mimic or control).	yes	more than 80% white	[60]
hereditary spastic paraplegia type 31	miR-140	*REEP1*	c.606+50G>A (A impairs G:U wobble base pairing)	*in silico*: miRNA target prediction program.	none	of European descent	[61]
hereditary spastic paraplegia type 31	miR-140	*REEP1*	c.606+43G>T (T impairs G:U wobble base pairing)	*in silico*: miRNA target prediction program.	none	of European descent	[61,62]
hereditary spastic paraplegia type 31	miR-691	*REEP1*	c.606+14C>T (unspecified)	*in silico*: miRNA target prediction program.	none	of European descent	[62]
breast cancer	miR-206	*ESR1*	rs9341070 C>T (T allows more effective binding)	*in vitro*: reporter gene assay in MCF-7 breast cancer cells (with pre-miR-206, miR inhibitor or *let-7* specific modified RNA).	none	—	[63]
hypertension	miR-155	*AGTR1*	rs5186 A>C (C impairs binding site)	*in vitro*: reporter gene assay in 293T cells (with miR mimic or *let-7c*).	yes	[64]	[64]
methotrexate resistance	miR-24	*DHFR*	rs34764978 C>T (T impairs binding efficiency)	*in vitro*: reporter gene assay in DG44 CHO cells (with miR mimic, miR inhibitor, positive or negative control).	none	—	[65]
childhood asthma	miR-148a, miR-148b, miR-152	*HLA-G*	rs1063320 C>G (G creates a new binding site)	*in vitro*: reporter gene assay in JEG3 cells (with miR mimic, positive or negative control).	yes	white Americans	[66]
arson or property damage	miR-96	*HTR1B*	rs13212041 [A/G] (G creates G:U wobble base pairing instead of Watson–Crick base pairing)	*in vitro*: reporter gene assay in HeLa cells (with miR mimic).	yes	white college students	[67]
colorectal cancer	miR-337, miR-582, miR-200a-5p, miR-184, miR-212	*CD86*	rs17281995 G>C (for miR-337, miR-582, and miR-200a-5p, C impairs binding efficiency; for miR-184 and miR-212, C increases binding efficiency)	*in silico*: miRNA target prediction program.	yes	from Czech republic	[68]
colorectal cancer	miR-612	*INSR*	rs1051690 G/A (unspecified)	*In silico*: miRNA target prediction program.	yes	from Czech republic	[68]
diarrhoea predominant irritable bowel syndrome	miR-510	*HTR3E*	rs56109847 (previously rs62625044) G>A (A impairs binding site)	*in vitro*: reporter gene assay in HEK293 and Colo320 cells (with miR precursor, miR inhibitor or negative control).	yes	British	[69]

### 2.2. Creation of new miRNA target sites by SNPs

#### 2.2.1. *MDM4* | miR-191 or miR-877-3p

Mdm2-like p53-binding protein (MDM4) is an oncoprotein that negatively regulates the p53 tumour suppressor protein [70]. It is well documented that overexpression of this protein leads to cancer development [70]. Recent studies suggested that the variation in the 3'-UTR of *MDM4* can lead to a decreased risk of various malignancies [42–47]. The occurrence of the C minor allele (SNP rs4245739 A>C) in the 3'-UTR of *MDM4* has been shown to decrease the risk of cancer, and delay the progression of metastasis and cancer-related death [42–47]. Numerous studies have shown that introduction of this C minor SNP creates a new binding site for miR-191 [42–46] and/or miR-887-3p [42,43,47], and this leads to a decreased level of MDM4 protein. Moreover, a recently conducted meta-analysis of 69 477 subjects (19 796 cases of nine various type of cancer and 49 681 controls) showed that the above-mentioned SNP correlates with a reduced overall risk of cancer [71].

#### 2.2.2. *ΔNp63* | miR-140-5p

p63 is another tumour suppressor protein belonging to the p53 family. Because of different promoters and alternative splicing, there are two major isoforms of *TP63*: *TAp63* (acidic transactivation domain present) and *ΔNp63* (no transactivation domain) [72]. Interestingly, *in vivo* experiments indicate that *TAp63* acts like a tumour suppressor gene, whereas *ΔNp63* is an oncogene [73–75]. Wang *et al*. [48] found that the SNP rs35592567 (C>T) in the 3′-UTR of *ΔNp63* has an impact on bladder cancer risk. Analysis showed that the T allele is correlated with a decreased risk of bladder cancer because miR-140-5p is able to bind to the 3'-UTR of *ΔNp63*. Overexpression of miR-140-5p in 5637 cells (urinary bladder grade II carcinoma cells) attenuated cell migration and invasion and inhibited cell proliferation [48].

#### 2.2.3. *HNF1B **|*** miR-214-5p and miR-550a-5p

Another example of a positive effect of a SNP on a disease risk is rs2229295 (C>A), which is located in the 3′-UTR of hepatocyte nuclear factor 1-beta *(HNF1B)* mRNA. This gene encodes a transcription factor known to be a regulator of growth and development in the pancreas [76]. Since HNF1B has a role in controlling hepatic insulin activity and glucose metabolism *in vivo* [77], Goda *et al.* [49] suggested that the rs2229295 SNP may correlate with susceptibility for type 2 diabetes mellitus (T2DM). Using luciferase reporter vectors, they demonstrated that the A allele constructs were regulated by two miRNAs: miR-214-5p and miR-550a-5p, whereas C allele constructs were not. Hence, the presence of A allele decreases HNF1B protein levels and has a protective effect against T2DM [49].

#### 2.2.4. *APOC3* and *APOA5* | miR-4271 and miR-485-5p

*APOC3* and *APOA5* are genes that encode apolipoprotein C3 and A5, respectively. Both of these proteins along with lipoprotein lipase (LPL) and apolipoprotein C2 (APOC2) are involved in triglyceride metabolism [50,51]. Hu *et al*. [50] demonstrated that decreased levels of APOC3 lead to lower triglyceride levels and reduce the risk of coronary heart disease (CHD). This is due to SNP (rs4225 G>T) found in the 3′-UTR of *APOC3*. When the T minor allele is present in the cell, miR-4271 is able to bind to the 3′-UTR of *APOC3,* and this leads to a decreased translation of APOC3. miR-4271, however, cannot bind to the variant containing the G major allele [50]. Similarly, *APOA5* c.*158C>T (rs2266788) is also associated with alterations in triglyceride metabolism and results in hypertriglyceridaemia [51]. In this case, the rare c.*158C *APOA5* allele creates a new functional binding site for miR-485-5p. Importantly, both miRNAs regulating *APOC3* and *APOA5* are endogenously expressed in the human liver, so if the SNP occurs, they may be involved in the regulation of triglyceride metabolism *in vivo*. However, both examples of SNPs and their impact on the risk of disease need further clarification, since different results have been obtained for different ethnic groups [50].

#### 2.2.5. *PLIN4* | miR-522

PLIN4 (perilipin 4) is a member of the perilipin family and these proteins coat the intracellular lipid storage droplets (LSD). PLIN4 has been proposed to promote uptake of free fatty acids from the blood to the LSD and is dependent upon the cell's nutritional status [78]. Meta-analysis of two populations of this gene, rs8887 (G>A), analysed with antropometric measurements, indicated that the two populations were different. Individuals with the A minor allele had an increased volume of visceral and subcutaneous adipose tissue, and higher BMI and weight compared to individuals with the G major allele [52]. This study reported that *PLIN4* is regulated by miR-522 only in the rs8887A variant. It is not yet clear, however, if the lower expression of *PLIN4* contributes to obesity because the results are conflicting [79,80].

#### 2.2.6. *FXN* | miR-124-3p

Reduced expression of the mitochondrial frataxin (FXN) protein has been postulated to play a role in Friedreich's ataxia (FRDA), an inherited neurodegenerative disease [81]. Lower levels of frataxin are due to GAA repeat expansion in the *FXN* gene [81]. Additionally, Bandiera *et al*. [53] have suggested that miR-124-3p regulates *FXN* expression *in vivo* only in FRDA patients. They identified seven SNPs in the 3′-UTR of *FXN* in children and adults diagnosed with FRDA. One of them, rs11145043 (G>T), permits miR-124-3p binding only when the T allele is present. Although miR-124-3p is highly expressed in the nervous system [82], it is overexpressed in FRDA patients [83], suggesting its role in FRDA. However, its influence on *FXN* needs further clarification.

### 2.3. Loss of miRNA target sites by SNPs

#### 2.3.1. *SCNA* | miR-34b

The α-synuclein *SCNA* gene polymorphism is considered a main risk for the common sporadic form of Parkinson's disease (PD; approx. 90% of all PD cases) [84]. α-Synuclein is a crucial protein that creates immunoreactive aggregates in Lewy-bodies, which are typical for Parkinson's disease patients' brains [85]. Studies have indicated that miR-34b targets the α-synuclein mRNA3′-UTR in two distinct sites and represses translation of this protein [86]. Importantly, in PD patients' brains, the level of miR-34b in the substantia nigra is decreased. Kabaria *et al.* [54] have identified a SNP, rs10024743 (T>G), in the 3′-UTR of α-synuclein, which is localized in the target site 1 of miR-34b. This SNP diminishes the miR-34b-mediated repression of α-synuclein levels due to disruption of the miRNA : mRNA association. However, this study was performed only on SH-SY5Y cells and its association with PD remains unclear [54].

#### 2.3.2. *PALLD |* miR-96 and miR-182

The *PALLD* gene encodes the actin-associated protein Palladin, whose expression correlates closely with the pathological cell motility characteristics of aggressive cancer cells. The expression level of Palladin in breast cancer patients is higher in invasive and malignant cancer cell types than in non-invasive and normal cell lines. The results suggest that Palladin promotes podosome formation, regulates the actin cytoskeleton via multiple pathways, participates in matrix degradation, and thus facilitates metastasis in breast cancer [87,88]. Gilam *et al*. [55] have reported that miR-96 and miR-182 reduce breast cancer cell migration and invasion by downregulating Palladin protein levels and that this process is disrupted by a SNP, rs1071738 (G < C), located in the 3′-UTR of the *PALLD* gene. This SNP is characterized by highest minor allele frequency (greater than 43%) and the alternate G allele is much more common than the ancestral minor C allele. If the C allele occurs in the binding site, the mRNA target sequence at the 3′-UTR of *PALLD* is fully complementary to the miR-96 and miR-182 seed regions, whereas the presence of the alternate G allele results in one mismatch. A significant decrease in Palladin levels is diminished by miR-96 and miR-182 expression (approx. 30% and approx. 70% reduction, respectively) in the presence of the C allele, but not in the presence of the G allele due to the disrupted miRNA:mRNA association. These findings suggest that although miR-96 and miR-182 may prevent breast cancer metastasis, the functional rs1071738 G variant abolishes this effect [55].

#### 2.3.3. *EFNB2* | miR-137

The *EFNB2* (ephrin-B2) gene encodes an ephrin, a protein tyrosine kinase that is involved in remodelling and the development of synaptic connections that are regulated by activated NMDA receptor. Ephrin-B2 is essential for the Reelin pathway controlling neuronal migration. Additionally, the activation of EFNB2 is crucial for rescuing the Reelin defect and disruption of this pathway is associated with schizophrenia [56,89]. Recently, a negative correlation between miR-137 and *EFNB2* expression was shown [56]. Importantly, the SNP rs550067317 (A>C) is located at the predicted target site of miR-137 in the 3′-UTR of *EFNB2*. The minor C allele of rs550067317 disrupts the formation of the typical stem-loop structure during base pairing of miR-137 with the predicted target sequence at the 3′-UTR, consequently reversing inhibition of *EFNB2* expression.

#### 2.3.4. *HIF1A* | miR-199a

The *HIF1A* gene encodes the HIF-1α protein (hypoxia-inducible factor 1), an oxygen dependent subunit and master transcriptional regulator of the mammalian cell response to oxygen deprivation, and is therefore important in both the cardiovascular and cancer fields. To date, numerous studies have demonstrated miRNA's role in regulation of HIF-1α levels [90–93]. Recently, a SNP (rs2057482 T>C) in the 3′-UTR of *HIF1A* located near the miR-199a binding site was identified [57,94]. The C allele of this variant has an increased frequency in pancreatic ductal adenocarcinoma patients and this CC genotype was characterized by a larger tumour size, shorter overall survival and a higher risk of this disease compared to CT and TT genotypes [57]. Additionally, the occurrence of the C allele was significantly associated with higher *HIF1A* mRNA and consequently upregulation of HIF1 levels, suggesting that this SNP impairs miR-199a : *HIF1A* binding [57].

#### 2.3.5. *DROSHA* | miR-27b

A very interesting example of a synonymous mutation that leads to the loss of an miRNA binding site is SNP rs10719 (T>C) located in the 3′-UTR of the *DROSHA* gene. The Drosha enzyme, a member of the RNAase III family, plays a critical role in miRNA biogenesis. It liberates the pre-miRNA stem-loop by cleavage of the longer pri-miRNAs in the nucleus [95]. In addition to this function, Drosha also influences cell proliferation and apoptosis [96]. Since overexpression of Drosha is observed in bladder cancer, this SNP is associated with an increased risk of bladder cancer [58]. Yuan *et al.* [58] reported that *DROSHA's* 3′-UTR contains a target site for miR-27b, while rs10719 (T>C) is located in close proximity to this site (46 bp downstream of the miR-27b binding site). They have postulated that rs10719T to C transition leads to weaker mRNA : miRNA association at the miR-27b target site and consequently to increased Drosha expression.

### 2.4. SNPs affecting the miRNA : mRNA interaction

#### 2.4.1. *FGF20* | miR-433

An example of another poly-miRTS related to PD was provided by Wang *et al.* [59], who reported a correlation between SNP (rs127202208 C/T) in the 3′-UTR of fibroblast growth factor 20 (*FGF20*) and the development of PD. *FGF20* is mainly expressed in substantia nigra and increases proliferation and promotes survival of dopaminergic neurons during the early stages of life. However, increased levels of FGF20 in the later stages of life enhance α-synuclein expression and can lead to the death of dopaminergic neurons [59]. miR-433, which is abundant in brain, was shown to downregulate the translation of FGF20, mainly because this reported SNP resides within the predicted binding site for miR-433. The allele C of this polymorphism represents a valid miRNA base pairing, whereas the T allele introduces a G : U wobble base pairing and consequently a mismatch, which affects the miRNA : mRNA interaction. However, this SNP does not eliminate the miRNA : mRNA binding, but attenuates it. This leads to increased FGF20 levels and indirectly to overexpression of α-synuclein. Importantly, the effect of this SNP on *FGF20* expression and its relationship to miR-433 levels were confirmed *in vivo* [59].

## 3. Conclusion

The discussed examples of poly-miRTSs strongly suggest that these SNPs can be crucial factors in developing human pathologies and could contribute to genetic diversity. As mentioned, roughly 180 000 SNPs in the human genome that are located in the 3'-UTR region were identified along with about 2600 mature miRNA sequences which are deposited in the mirBase (v. 21), suggesting that a large number of these SNPs may introduce miRNA-binding changes. Furthermore, the recent development of deep sequencing techniques and advanced database/software tools like miRSNP and PolymiRTS Database 3.0 (see table 2 for complete list) allows researchers to initially access potential poly-miRTSs. Hence, in the near future, we can expect growing numbers of studies linking poly-miRTSs to human diseases.

**Table 2.** Current software and databases dedicated for poly-miRTS studies.

name	website	applications	refs
polymiRTS Database 3.0	http://compbio.uthsc.edu/miRSNP/	SNPs and INDELs in miRNA target sites identified from various experiments, predicted miRNA target sites, miRNA seeds	[97]
miRSNP	http://bioinfo.bjmu.edu.cn/mirsnp/search/	SNPs in predicted miRNA target sites	[98]
microRNA-related single nucleotide polymorphism	http://www.bioguo.org/miRNASNP/	SNPs in human pre-miRNAs, in human miRNA flanks, in miRNAs of other species, target gain/loss by SNP in miRNA seed or in target 3′-UTR	[21]
miRdSNP	http://mirdsnp.ccr.buffalo.edu/	disease-associated SNPs and microRNA target sites on 3′-UTRs of human genes	[99]
ImiRP (illegitimate microRNA predictor)	http://imirp.org/	mutations in predicted miRNAs target sites	[100]

In 2008, Sethupathy & Collins [17] provided criteria for assigning SNPs as poly-miRTSs involved in human diseases that include: (i) functional (preferably *in vivo*) experimental validation of SNPs related to differential mRNA targeting; (ii) genetic testing of the association with the disease that takes into account the effects of population stratification; and finally (iii) mechanistic testing underlying the mechanism by which poly-miRTSs contribute to the disease [17]. Few current studies satisfy all these criteria (table 1), while the majority of them rely on population correlation effects and *in silico* modelling only, ignoring the necessity of the mechanistic approach. Importantly, commonly used methods to confirm differential miRNA : mRNA binding, *in vitro* luciferase reporter constructs and miRNA overexpression often do not consider the physiological miRNA levels *in vivo*. However, miRNA physiological levels are often undergoing dynamic changes due to epigenetic factors [101], and thus they can affect the verification of the poly-miRTS disease-related mechanisms. The luciferase-based reporter assays are usually performed in artificial cancer cell lines that permit easy AgoMiR (mimic) delivery, and are often characterized by low endogenous miRNA levels. The latter inhibits endogenous miRNAs from degrading the reporters prior to the miRNA overexpression. Importantly, the miRNA overexpression in these systems is often a hundred fold higher than *in vivo* conditions. Hence, in the case of validation of new target sites created by poly-miRTS, this experimental model may lead to false positive results, since it cannot differentiate between weak and strong binding to the targets. The vector-based miRNA expression system that provides inducible and scalable control over miRNA levels may provide more solid verification of potential miRNA : mRNA binding [102].

Recently, the development of morpholino-based target protector technology provides an elegant tool to test the functionality of novel potential miRNA : mRNA interactions that mimics physiological conditions [103,104]. Target protectors bind to specific target mRNA sequences and block miRNA access, however without triggering an RNAi response [105]. Hence, target protectors allow blocking the miRNA-mediated suppression of a specific target mRNA [105]. Importantly, these modified oligonucleotides can be used to evaluate the significance of miRNA : mRNA interactions in the context of physiological miRNA levels.

Furthermore, often changes in a gene's mRNA level are not reflected in its protein levels [106]. Hence, the studies of miRNA SNP-affected targets should be always accompanied by monitoring protein levels in cell lines related to the disease. Finally, although in research models usually one miRNA and one target are considered, the single miRNA usually is predicted to bind hundreds of target mRNAs, and have multiple effects on cellular metabolism. Hence, studying the mechanism of poly-miRTS involvement in human diseases requires verification that the miRNA effects are a result of indirect disease-related targets. Although this possibility cannot be totally eliminated, following genome-wide effects of specific miRNA modulation (with next generation sequencing) can support direct miRNA : mRNA interactions.

The most convincing and final criterion for linking poly-miRTSs to disease is establishing the disease-related mechanisms of differential miRNA binding. Taking into account complexity of a potential SNP effect on miRNA : mRNA pairing, this can be challenging. Nevertheless, the recent development of targeted genome editing tools (like CRISPR/Cas9 systems) allows one to make efficient, precise and targeted changes to the genome of the living cells, and opens novel possibilities to overcome this limitation [107]. Sadly, to date, no study has been reported in which targeted genome editing was applied in order to validate poly-miRTSs.

Analysing the specific effects of homozygotic and heterozygotic SNPs in both *in vitro* and *in vivo* disease models could provide the critical proof for the role of and frequency that poly-miRTSs occur in human diseases.
